# Impact of Low-Frequency Alternating Electromagnetic Fields on Postharvest Preservation of Satsuma Mandarins

**DOI:** 10.3390/foods14132307

**Published:** 2025-06-29

**Authors:** Qunhui Dong, Jiamiao Hu, Yihui Lu, Yujin Cao, Shaoling Lin

**Affiliations:** College of Food Sciences, Fujian Agriculture and Forestry University, Fuzhou 350002, China; 12409019065@fafu.edu.cn (Q.D.); jiamiaohu@fafu.edu.cn (J.H.); luyihui17349662280@163.com (Y.L.); 15059806533@163.com (Y.C.)

**Keywords:** alternating electromagnetic field, citrus fruits, satsuma mandarins, postharvest storage, electromagnetic field intensity

## Abstract

Low-frequency alternating electromagnetic fields (LF-AEMF) represent an innovative processing technology with significant potential for extending the shelf life of fruits and vegetables by modulating key physiological processes. In this study, the impact of the LF-AEMF intensities (1300, 1800, and 2500 V) on the postharvest preservation of satsuma mandarins was evaluated. Compared to the control group, the LF-AEMF-treated samples exhibited reduced weight loss (0.62% vs. 2.11%), respiration rate (32.73 vs. 40.08 mg/kg·h), and malondialdehyde (MDA) content (40.80 vs. 34.87 nmol/g) after 40 days of storage. In addition, LF-AEMF treatment also effectively preserved titratable acidity (TA) (0.34% vs. 0.30%), vitamin C (Vc) content (7.77 vs. 7.05 g/100 g), and phenylalanine ammonia-lyase (PAL) activity (79.757 vs. 62.395 U/g). E-nose analysis and low-field NMR further revealed that the application of LF-AEMF effectively facilitated the superior preservation of the intrinsic flavor profile of the satsuma mandarins and mitigated the loss of free water within the fruit. Overall, this research provides valuable insights for the potential application of LF-AEMF in extending the storage life of citrus fruits, which may also be applicable to other seasonal fruits and vegetables that require long-term storage.

## 1. Introduction

Citrus fruits, classified within the genus *Citrus* of the *Rutaceae* family, are extensively cultivated and widely consumed. Among these, oranges, lemons, limes, grapefruits, and satsuma mandarins represent the most commercially important species [1]. These fruits are an abundant source of vitamin C (Vc), simple sugars, and contain a variety of bioactive compounds such as dietary fiber and flavonoids, which exhibit notable antioxidant properties and contribute to numerous health benefits [2,3]. The global popularity of citrus fruits is also attributed to their high yields, aromatic fragrance, and appealing flavor. According to data from the World Citrus Organization, citrus cultivation spans over 140 countries, with global production approaching 160 million metric tons annually. China is the leading producer of citrus fruits worldwide, followed by Brazil, India, and Mexico.

Nonetheless, citrus fruits have a relatively short postharvest life and present significant challenges in preservation, making their storage difficult. It has been reported that the loss rate of citrus fruits can be nearly 20% within the fruit industry chain (production, postharvest handling, storage, circulation, and consumption), of which 1/3 of the loss is related to the circulation and storage stages [4,5]. During storage, citrus fruits are prone to weight loss, softening, and flavor deterioration, largely due to postharvest physiological and biochemical processes such as respiration, metabolism, and transpiration [6]. Moreover, factors such as temperature, oxygen, microorganisms, and enzyme activity can profoundly influence the postharvest physiological changes of fruits and vegetables. Given the significance of these factors, various preservation techniques, such as temperature control, UV irradiation, biofilm and coating applications, and modified atmosphere packaging, have been employed to mitigate postharvest deterioration and extend the shelf life of fruits and vegetables by modulating these external and internal factors [7,8,9]. For instance, Ning et al. [10] discovered that low-temperature storage effectively inhibited the production rates of malondialdehyde (MDA) and hydrogen peroxide (H_2_O_2_) in melons by inducing the expression of antioxidant enzymes. Similarly, Quan et al. [11] reported that application of chitosan film in combination with microcapsule technology significantly delayed weight loss in citrus fruits. Additionally, the research by Li et al. [6] has confirmed that the chitosan-based composite coatings incorporated with polyethoxylated flavonoids (PMFs)-loaded citral can be used to preserve citrus fruits by inhibiting the proliferation of *Penicillium* fungi and maintaining the antioxidant enzyme activity. However, many of these methods involve complex procedures, may contribute to environmental pollution, or require specialized equipment. As a result, there remains a pressing need for a preservation technique that is cost-effective, environmentally sustainable, and highly efficient [12].

As a non-thermal processing technology, low-frequency alternating electromagnetic fields (LF-AEMF) treatment is characterized by its safety and efficiency, ease of operation, and environmentally friendly. When subjected to an alternating electromagnetic field, water molecules within cells are aligned, thereby reducing the internal water flow in food tissues. This technology also mitigates the damage caused by ice crystals to cells during the freezing process of food, inhibits the activity of oxidative enzymes, and consequently extends the shelf life of fruits and vegetables [13,14]. Indeed, a range of studies have shown that electromagnetic field technologies could profoundly influence the quality of foods during storage and effectively maintain the freshness of agricultural products. For instance, Mahsa et al. [15] conducted research on the thawing rate and physicochemical properties of rainbow trout fillets using alternating magnetic fields (AMF). The results indicated that compared with refrigerator thawing, treatment with AMF significantly reduced both the thawing time and the thawing rate. Yang et al. [16] also found that AMF could reduce weight loss and maintain the activity of antioxidant enzymes in cherry tomatoes. Similarly, Xiong et al. [17] reported positive effects of alternating current electric fields on cabbage and spinach. Moreover, Faridnia F et al. [18] showed that pulsed electric fields can affect the microstructure of beef, improving its water retention ability and quality. In addition, Zhou et al. [19] employed low-voltage electric fields (LVEF) for the storage of lychee and discovered that LVEF could effectively reduce the activity of polyphenol oxidase and peroxidase, successfully delaying the browning of the fruit peel. Variations in electromagnetic fields can affect the distribution of charges within tissues, which in turn can disrupt normal energy transfer and material exchange processes. Different forms and intensities of electromagnetic fields have different effects on the tissues of different fruits and vegetables, meaning that the optimal electric field intensity for effective preservation may vary depending on the type of product. Liu et al. [12] applied varying intensities of electric field treatments to cherry tomatoes and confirmed that there are significant differences in their preservative effects.

To date, the use of electromagnetic field technologies for citrus fruit preservation remains underexplored. This study takes satsuma mandarins as the experimental subject and quantitatively evaluates the effects of applying LF-AEMF at different intensities (1300, 1800, and 2500 V) on the preservation of the fruit. The aim is to explore the potential application of electromagnetic field-based preservation technologies for citrus fruits. Moreover, it may also offer a vital reference for employing LF-AEMF technology in the preservation of other fruits and vegetables that necessitate long-term storage.

## 2. Materials and Methods

### 2.1. Materials

**Figure 1:**
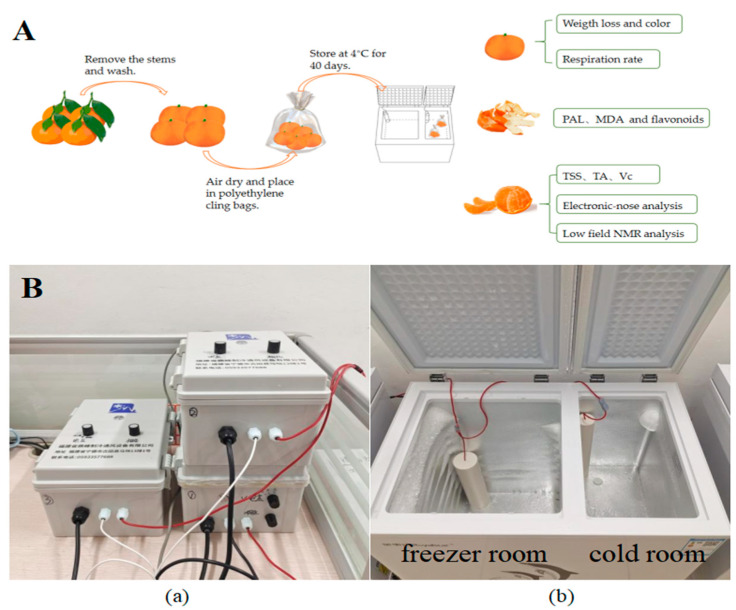
(**A**) Schematic diagram of experimental process. (**B**) Photograph of LF-AEMF generating device. Voltage controller (**a**) and refrigerator (**b**).

In early January 2025, satsuma mandarins at commercial maturity were meticulously harvested from an orchard located in Fuzhou city (Fujian, China). Subsequently, the fruits without mechanical damage and disease were carefully selected based on their weight (50–60 g) and uniformity in color. Following harvest, these fruits were transported to the laboratory. The fruits were gently rinsed with tap water and allowed to air-dry. They were then individually wrapped in polyethylene cling bags and pre-stored at a temperature of 4 ± 1 °C for 24 h. The fruits were randomly allocated into four distinct groups, each comprising 30 samples: (1) CK group (4 ± 1 °C, no treatment); (2) L-LF-AEMF group (4 ± 1 °C with LF-AEMF treatment at 1300 V); (3) M-LF-AEMF group (4 ± 1 °C with LF-AEMF treatment at 1800 V); and (4) H-LF-AEMF group (4 ± 1 °C with LF-AEMF treatment at 2500 V). All samples were kept at a constant temperature of 4 ± 1 °C for 40 days, and the LF-AEMF was applied to the treatment group during storage. The physical and chemical properties of the samples were assessed at 5-day intervals. The experimental process for satsuma mandarins is shown in Figure 1A.

### 2.2. Experimental Device

In this study, the satsuma mandarins were stored in the refrigerator (KCD-158D, Hefei Rongshida Electronic Technology Co., Ltd., Hefei, China) equipped with an electromagnetic field generator (DF-DCBX-001, Dingfeng Refrigeration and Ventilation Equipment Co., Ltd., Ningde, China). The internal dimensions of the cold room were 220 × 385 × 595 mm, and the temperature is maintained at 4 ± 1 °C. The schematic diagram of the LF-AEMF generating device is shown in Figure 1B. The output voltage was regulated by the electric field regulator until the voltages reached 1300, 1800, and 2500 V.

### 2.3. Weight Loss and Color Measurements

At each selected time interval, five satsuma mandarins were randomly selected from each experimental group, individually labeled as 1, 2, 3, 4, and 5, and subsequently utilized for the measurement of weight loss. The weight of each individual fruit was precisely determined using electronic scales. The weight loss rate was then calculated utilizing the following formulas [6]:

(1) $$\mathrm{Weight}\;\mathrm{loss}\;(\%)=\frac{W_{0}-W_{d}}{W_{0}}\times 100$$

where W_0_ is the initial weight (g), and W_d_ is the final weight of citrus fruits (g).

The color difference of the fruit skin was assessed using a 3NH colorimeter (Guangdong SanEnshi Technology Co., Ltd., Guangzhou, China). Specifically, the measurements were performed at two points symmetrically positioned relative to the equatorial plane of the fruit. The a* value, which reflects the red–green axis in the color space, was recorded [20].

### 2.4. Measurement of Respiration Rate

Three samples were selected from each group and were placed into a hermetically sealed glass container with a capacity of 2 L. The concentration of carbon dioxide within the container was continuously monitored using the JM-HX20 fruit and vegetable respirometer (Shanghai Precision Instrumentation Co., Ltd., Shanghai, China) to assess the respiration intensity of the samples. The respiration rate, quantified by the production of CO_2_, was expressed in units of mg/kg·h.

### 2.5. Total Soluble Solids (TSS), Titratable Acidity (TA), and Vitamin C (Vc) Content

Three samples were randomly taken from each group for the determination of TSS, TA, and Vc content. The TSS contents were ascertained using a DLX-ARTT050 refractometer (Delixi Electric Ltd., Hangzhou, China) and were presented as °Brix.

The TA was determined following the method described by Naeem et al. [21], with minor adjustments. Specifically, a 10.0 g sample of satsuma mandarin was homogenized with 50 mL of distilled water that had been boiled and subsequently cooled. The mixture was heated to 70 °C for 15 min in a water bath, then cooled under running water. After adjusting the volume to 100 mL with distilled water, the sample was centrifuged. An appropriate volume of the filtrate was treated with 2 drops of 1% (*w*/*v*) phenolphthalein. Titration was then carried out using a 0.1 M NaOH standard solution until a pink coloration was observed. The volume of NaOH consumed was recorded, and TA content was calculated based on citric acidity equivalence.

(2) $$\mathrm{Titratable}\;\mathrm{acidity}\;\mathrm{content}\;(\%)=\frac{CVKV_{0}}{MV_{1}}\times 100$$

where C is the titration concentration of sodium hydroxide (mol/L); V represents the volume of sodium hydroxide solution consumed by the titration filtrate (mL); K is the conversion coefficient, 0.064 g/mmol; V_0_ is the total volume of sample extract (mL); M is the mass of sample (g); and V_1_ is volume of filtrate extracted during titration (mL).

According to the method of Quan et al. [11], the absorbance values of the satsuma mandarin at a wavelength of 243 nm were measured utilizing a UV spectrophotometer (Thermo Fisher Scientific, Madison, WI, USA). Subsequently, a standard curve was constructed correlating absorbance to the concentration of Vc, and the calculation was based on the following equation:

(3) $$\mathrm{Vc}\;\mathrm{content}\;(\mathrm{mg}/g)=\frac{C}{V}\times 25$$

where C is the concentration of Vc corresponding to the sample solution according to the standard curve (μg/mL); V is the volume of the sample solution (mL).

### 2.6. Phenylalanine Ammonia-Lyase (PAL) Activity, Malondialdehyde (MDA), and Flavonoids Content

Determination of PAL activity: From each sample, precisely weigh out 0.1 g of citrus peel and subject it to ice bath homogenization with 0.1 g of the extraction solution provided in the PAL assay kit (Beijing Solarbio Science & Technology Co., Ltd., Beijing, China). Subsequently, in accordance with the manufacturer’s instructions, add the appropriate solutions and measure the absorbance of the supernatant at 290 nm. The unit of PAL activity is U/g.

Determination of MDA content: First, 0.1 g of citrus peel was mixed with the extraction solution included in the MDA assay kit (Beijing Solarbio Science & Technology Co., Ltd., Beijing, China) and then ground on ice. Then, the other reagents in the kit were mixed and added to the sample solution according to the reagent vendor’s protocol. The mixture was heated at 100 °C for 60 min, cooled in an ice bath, and centrifuged. Finally, the absorbance of the sample was determined at 532 nm and 600 nm, and the MDA content was expressed in nmol/g.

Determination of flavonoids content: Citrus peels were dried and pulverized, then 0.1 g of the sample powder was weighed and added to the extraction solution in flavonoids content assay kit (Beijing Solarbio Science & Technology Co., Ltd., Beijing, China), and the sample solution to be tested was prepared by ultrasonic extraction. Subsequently, the absorbance of the sample was measured at 470 nm according to the method of the kit. The unit of flavonoid content is mg/g.

### 2.7. Electronic Nose Analysis

The sensory evaluation of the flavor of satsuma mandarins was conducted in accordance with Penke’s method, with minor modifications [22]. Satsuma mandarins were ground to make a homogenate, about 10 mL of citrus juice was taken and sealed in a headspace bottle, and then equilibrated for 30 min at room temperature. Subsequently, the volatile compounds were analyzed using a PEN3 electronic nose device (AIRSENSE Analytics GmbH, Schwerin, Germany). The operational parameters of the electronic nose were set as follows: a sample interval of 1 s, an air flow rate of 500 mL/min, a measurement time of 120 s, a flush time of 120 s, and a testing temperature maintained at room temperature. The performance of the electronic nose sensors is detailed in Table 1, presented below.

### 2.8. Low-Field NMR Analysis

A single segment was excised from each sample and positioned within a cylindrical coil for analysis. The LF-NMR (PQ001, Niumag Analytical Instrument Co., Ltd., Shanghai, China) was used to determine the water distribution in the samples. The apparent transverse relaxation time (T_2_) spectra were obtained via iterative inversion techniques. The pulse sequence parameters were configured as follows: the repetition time was set as 5000 ms, the echo time was established at 0.5 ms, and the number of echoes was 15,000.

### 2.9. Statistical Analysis

All experimental procedures were conducted in triplicate to ensure data reliability. The results are presented as the mean ± standard deviation (SD). Data collection and graphical representation were accomplished using Excel and Origin 2021 software. The statistical analysis was conducted via one-way analysis of variance (ANOVA), with significant differences among treatment groups determined by Duncan’s multiple-range test at a significance level of *p* < 0.05.

## 3. Results and Discussion

### 3.1. Impact of the LF-AEMF on the Weight Loss and Color Changes of Satsuma Mandarins During Storage

**Figure 2:**
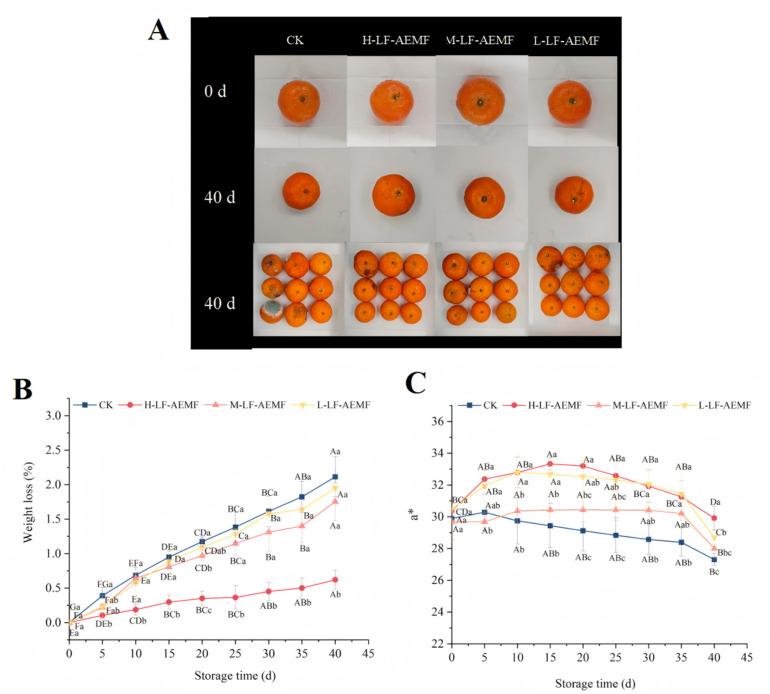
Appearance (**A**), weight loss (**B**), and a* values (**C**) of satsuma mandarins from different treatment groups during storage. CK (4 ± 1 °C, 40 d); L-LF-AEMF (4 ± 1 °C, 40 d, 1300 V); M-LF-AEMF (4 ± 1 °C, 40 d, 1800 V); H-LF-AEMF (4 ± 1 °C, 40 d, 2500 V). Lowercase letters (a–c) present significant differences among different treatments; capital letters (A–G) indicate significant differences among different storage times (*p* < 0.05).

Weight loss is a critical indicator reflecting the quality deterioration of fruits and vegetables during storage [23]. The rate of weight loss in fruits increased progressively with prolonged storage time (Figure 2B), a phenomenon typically ascribed to the fruits’ inherent respiration, transpiration, and senescence processes. However, the application of LF-AEMF effectively mitigated this progressive increase in weight loss. This phenomenon is in accordance with the results of low-field nuclear magnetic resonance (LF-NMR), indicating that the application of LF-AEMF restricts the dynamic behavior of water molecules within the tissue of satsuma mandarins, thereby reducing the loss of free water and contributing to the maintenance of the weight of satsuma mandarins. Notably, the H-LF-AEMF group demonstrated the most significant reduction in weight loss (*p* < 0.05), followed by the M-LF-AEMF and L-LF-AEMF groups. The substantial reduction in weight loss observed in satsuma mandarins subjected to LF-AEMF treatment is consistent with findings reported by Zhou et al. [19] for litchi fruits, who also observed an improvement in weight retention following LF-AEMF treatment when compared to the untreated control group.

The a* value represents the color shift on the red–green axis, with a positive number representing a reddish bias and a negative number representing a greenish bias. As illustrated in Figure 2C, the CK group exhibited a consistent downward trend in the a* value. In contrast, the LF-AEMF-treated samples displayed an initial rise, followed by a subsequent decline, yet throughout all measured time points, the a* values remained higher than those of the CK group. The a* value signifies that the LF-AEMF treatment resulted in satsuma mandarins with a more pronounced red color, which is likely to be more visually appealing. Indeed, a previous study has reported that, compared with the control group, the combination of modified atmosphere packaging (MAP) and 1-methylcyclopropene (1-MCP) treatment can maintain a higher a* value of “Hongmeiren” citrus, indicating its potential to enhance citrus preservation.

### 3.2. Impact of the LF-AEMF on the Respiration Rate of Satsuma Mandarins During Storage

**Figure 3:**
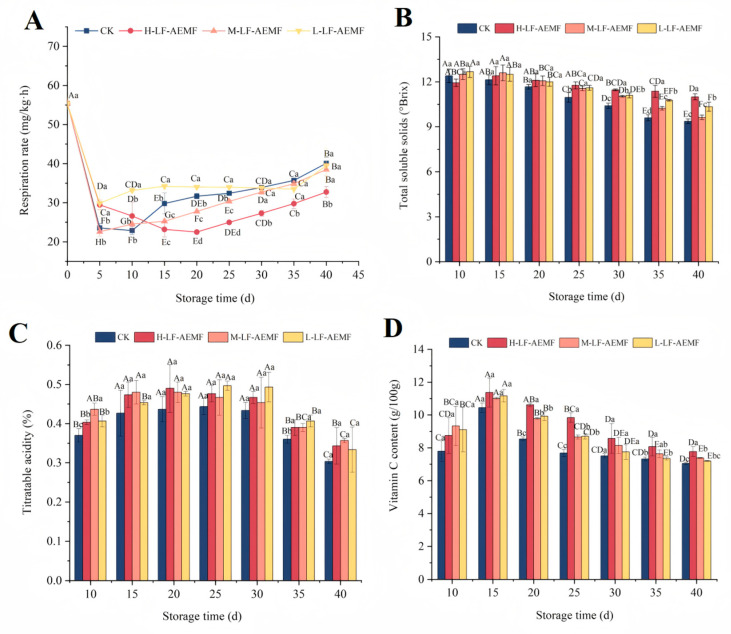
Changes in respiratory strength (**A**), total soluble solids (**B**), titratable acidity (**C**), and Vc content (**D**). CK (4 ± 1 °C, 40 d); L-LF-AEMF (4 ± 1 °C, 40 d, 1300 V); M-LF-AEMF (4 ± 1 °C, 40 d, 1800 V); H-LF-AEMF (4 ± 1 °C, 40 d, 2500 V). The data are the average of three replicates ± standard errors. Lowercase letters (a–d) present significant differences among different treatments; capital letters (A–H) indicate significant differences among different storage times (*p* < 0.05).

As depicted in Figure 3A, the respiration rate of the fruits across all treatment groups exhibited an initial decline followed by a subsequent rise. Notably, on the fifth day, there was a marked decrease in respiration rate across all groups, which can be attributed to the environmental stress encountered. After 15 days, a gradual increase in respiratory rate was observed in the fruits of all groups. However, the H-LF-AEMF group demonstrated a significantly lower respiration rate compared to the CK group (*p* < 0.05). Additionally, the M-LF-AEMF group experienced a significant reduction (*p* < 0.05) from the 15th day to the 20th day. The respiration of fruits during storage is a critical physiological process that not only depletes the organic nutrients within the fruit but also significantly impacts the sensory attributes and shelf life of the fruit [24]. The LF-AEMF treatment has a more pronounced effect on reducing the respiration rate of fruits. This effect may be partially attributed to the fact that the LF-AEMF causes a more compact arrangement of the phospholipid bilayer in the cell membrane. Polar charged ions within the cell membrane, such as K⁺ and Ca²⁺, induce changes in membrane voltage. These changes lead to a decrease in the permeability and electrical conductivity of the cell membrane, thereby inhibiting the respiration process [25]. In addition, Ji et al. [26] also suggested that magnetic field treatment may compromise the functionality of respiratory enzymes, thereby leading to a decrease in respiration rate. This may also be an underlying mechanism of the decreased respiration rate observed in LF-AEMF-treated samples.

### 3.3. Impact of the LF-AEMF on the Fruit Quality Indicators TSS, TA, and Vc Contents of Satsuma Mandarins During Storage

The TSS, TA, and Vc contents are key indicators of fruit maturity and play a crucial role in determining their flavor profile. Our experimental findings revealed a progressive decline in TSS content in satsuma mandarins during storage (Figure 3B), which is attributed to the sugar conversion processes occurring during ripening and post-ripening stages. Notably, the TSS concentration in the pulp of LF-AEMF-treated groups consistently exceeded that of the CK group throughout the storage period. Moreover, the LF-AEMF treatment exerted a significant influence on the TSS content of satsuma mandarins, particularly after day 25 of storage. By the end of the storage period, the TSS content in the CK, H-LF-AEMF, M-LF-AEMF, and L-LF-AEMF groups was 9.37%, 11%, 9.63%, and 10.33%, respectively. This suggests that the LF-AEMF treatment may delay the consumption of sugars in the citrus pulp. This effect is likely attributable to the ability of the LF-AEMF to reduce the respiration rate and overall metabolic activity of the fruit, thereby slowing the degradation of TSS over the storage period [27]. Similar results were reported by Yang et al. [28], who applied DENBA^+^ technology to strawberry preservation and demonstrated that the technology could delay the process of nutrient consumption in strawberry fruit.

TA is a crucial substrate for fruit respiration and also the primary source for synthesizing energy ATP [29]. Its content typically decreases with the extension of storage time. Our observations indicate that as the duration of storage extends, the TA levels in all groups increase and then decrease. Throughout the entire storage period, the patterns of TA change among the various treatment groups were quite comparable, and the TA content consistently higher than in the CK group. However, this difference in TA content tended to decrease in the later stages of storage.

Vc is a key bioactive compound that determines the nutritional quality of agricultural products. It is highly susceptible to degradation via both enzymatic and non-enzymatic pathways during storage. The reduction in Vc content is a significant indicator of quality deterioration during the storage period of citrus fruits. Therefore, the maintenance of Vc content serves as a crucial indicator for the effective preservation of fruits. For instance, the study by Quan et al. [11] has demonstrated that, compared to the control group, the application of chitosan film in combination with microcapsule technology can effectively inhibit the degradation of Vc content in citrus fruits during storage. In our study, during the initial 15 days of storage, an elevation in Vc content was observed in both the CK and LF-AEMF-treated satsuma mandarins, culminating in peak concentrations of 10.45, 11.37, 11.01, and 11.17 g/100 g, respectively (Figure 3D). Subsequently, a downward trend in Vc levels was noted for the following storage duration. It is worth noting that under the same storage conditions, the Vc content in fruits treated with LF-AEMF was always higher than that in the CK group, and the H-LF-AEMF group was most effective in maintaining Vc content (*p* < 0.05).

Notably, as the storage time extends, the respiration and metabolic activities of the fruit accelerate the degradation of TA and Vc. The LF-AEMF and low-temperature treatment may modulate the internal physiological and metabolic processes of the fruit, thereby inhibiting respiration and the activity of relevant enzymes, which in turn can slow down the degradation rate of TA and Vc [12]. This helps maintain the original flavor of the fruit and delay its aging process.

### 3.4. Impact of the LF-AEMF on the MDA Content, PAL Activity, and Flavonoids Content of Satsuma Mandarins During Storage

**Figure 4:**
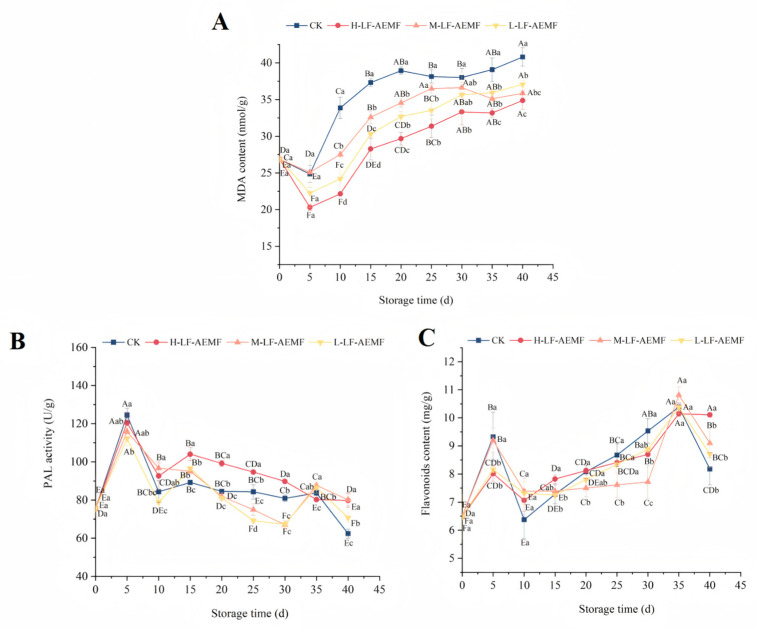
Changes in MDA content (**A**), PAL activity (**B**), and flavonoids content (**C**) of fruit peel during storage in different treatment groups. CK (4 ± 1 °C, 40 d); L-LF-AEMF (4 ± 1 °C, 40 d, 1300 V); M-LF-AEMF (4 ± 1 °C, 40 d, 1800 V); H-LF-AEMF (4 ± 1 °C, 40 d, 2500 V). Different letters present significant differences among treatments at the same storage time (*p* < 0.05).

During the postharvest senescence process, MDA serves as the end product of lipid peroxidation and is a critical indicator of membrane system damage [30,31]. Observations indicate that MDA content exhibits a declining trend during the initial phase of storage, which may be attributed to the low-temperature storage conditions that suppress the respiratory metabolism of the fruits and consequently slow down the ripening process. This reduction in metabolic activity leads to a decrease in MDA content. The concentration of MDA in the peel of satsuma mandarins, as depicted in Figure 4A, showed an increasing trend in all treatment groups after the 10th day of storage. Similar observations were previously documented by Xu et al. [32] in their study using a static magnetic field to treat freshly cut lotus root. The rate of MDA accumulation in fruits subjected to LF-AEMF treatment was significantly decelerated in comparison to those in the CK group, with the H-LF-AEMF group demonstrating the most pronounced reduction (*p* < 0.05). At the end of the storage (40 d), the MDA contents of CK, H-LF-AEMF, M-LF-AEMF, and L-LF-AEMF treatments were 40.80, 34.87, 35.83, and 37.06 nmol/g, respectively. Notably, MDA levels indicate cell oxidative damage, which is often determined by the synergistic interactions among various factors, such as respiration rate, microorganisms, free radicals, and antioxidant enzymes [6,33]. The application of LF-AEMF exerts an influence on several homeostatic mechanisms that contribute to the preservation of the product. These mechanisms include the suppression of respiration intensity, inhibition of microbial growth, reduction of reactive oxygen species accumulation, and mitigation of cell wall degradation. Consequently, LF-AEMF treatment effectively alleviates the process of membrane lipid peroxidation in citrus fruits during storage.

The phenylpropanoid pathway constitutes one of the three principal secondary metabolic pathways within the plant kingdom, with PAL serving as the rate-limiting enzyme in this sequence. PAL plays a vital role in both the growth and development of plants as well as their adaptive responses to various environmental stressors [34]. As illustrated in Figure 4B, the peak activity of PAL in the satsuma mandarins across the control (CK), H-LF-AEMF, M-LF-AEMF, and L-LF-AEMF groups was recorded at 124.458, 120.443, 115.91, and 112.162 U/g, respectively, on the fifth day of storage. During the storage period, it was observed that the peels of the samples in the H-LF-AEMF group exhibited significantly elevated PAL activity when compared to the control group (*p* < 0.05). This phenomenon is less pronounced in the M-LF-AEMF and L-LF-AEMF groups, probably because the changes in PAL activity during storage are influenced by a variety of factors, such as temperature, fruit hormone levels, stress resistance, and pathogen infection. Therefore, a higher intensity of LF-AEMF treatment is required to show statistically significant changes.

The biosynthesis of flavonoids in plants is mediated through the phenylpropanoid pathway, a process that is facilitated by an array of enzymes including PAL, cinnamic acidity 4-hydroxylase (C4H), and 4-coumarate CoA ligase (4CL) [35]. On the 5th and the 35th days, the concentrations of flavonoids compounds in the four different groups reached their peaks. These fluctuations in flavonoids levels corresponded with the variations observed in PAL activity, suggesting a correlation between PAL activity and flavonoids synthesis during these time points. Nonetheless, during the mid-point of the storage period, a decrease in PAL activity was noted, whereas the flavonoids content increased. This discrepancy suggests that the alterations in the activities of flavonoids-related metabolic enzymes and the levels of flavonoids themselves do not exhibit a perfectly congruent relationship.

### 3.5. Impact of the LF-AEMF on the E-Nose Signal of Satsuma Mandarins During Storage

**Figure 5:**
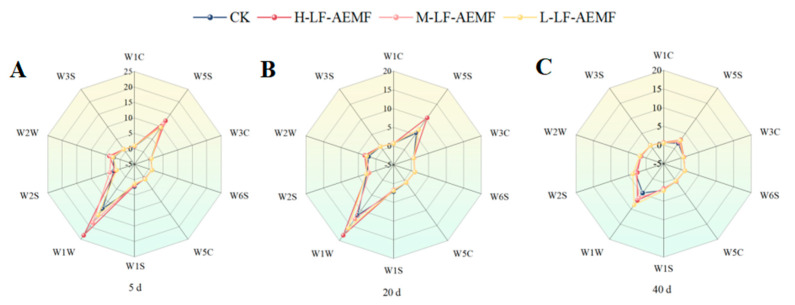
The changes in flavor of satsuma mandarins in different treatment groups on the 5th (**A**), 20th (**B**), and 40th (**C**) days. CK (4 ± 1 °C); L-LF-AEMF (4 ± 1 °C, 1300 V); M-LF-AEMF (4 ± 1 °C, 1800 V); H-LF-AEMF (4 ± 1 °C, 2500 V).

As illustrated in Figure 5 and detailed in Table 1, the analysis of citrus juice samples revealed a pronounced response to the sensors W5S (sensitive to nitrogen oxides), W1W (sensitive to sulfides), and W2W (sensitive to aromatic compounds). In contrast, the remaining sensors elicited a relatively weak reaction from the samples. These observations are consistent with those of a Chinese scholar, who reported that sensor W5S exhibits sensitivity to volatile compounds in satsuma mandarins, while sensors W6S and W3S demonstrate a lack of sensitivity towards the aromatic substances of these fruits. Notably, the treatment group consistently had stronger signals for W5S, W1W, and W2W until 20 days of storage. This observation suggests that the treatment group may have contributed to the maintenance of a more favorable odor profile in the fruit. As the duration of storage extends, there is a marked decrease in the intensity of all measured signals, and the disparities in signal values among the various treatment groups diminish over time. As the storage time was extended, the intensity of all measured signals decreased significantly, indicating that the volatile compounds in citrus juice gradually diminished, especially nitrogen oxides and sulfur compounds. Moreover, the disparities in signal values among the various treatment groups diminish over time.

### 3.6. Impact of the LF-AEMF on Low-Field NMR Signal of Satsuma Mandarins During Storage

**Figure 6:**
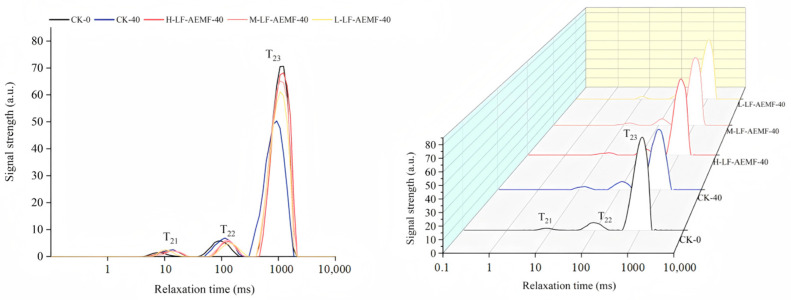
T_2_ inversion spectra of the horizontal relaxation time for satsuma mandarins in all groups on the 0th day and the 40th day. CK-0 (4 ± 1 °C, day 0); CK-40 (4 ± 1 °C, day 40); L-LF-AEMF-40 (4 ± 1 °C, day 40, 1300 V); M-LF-AEMF-40 (4 ± 1 °C, day 40, 1800 V); H-LF-AEMF-40 (4 ± 1 °C, day 40, 2500 V).

Low-field NMR techniques can be used to detect the state of water bound to food components and the moisture distribution in food products [36]. Figure 6 displays the transverse relaxation time spectra (T_2_ spectra) under different treatments. These spectra exhibited three distinct peaks, a finding that aligns with the reports by Zhu et al. [37]. The peaks are sequentially denoted from left to right as T_21_ (1.0–10.0 ms), T_22_ (10.0–100 ms), and T_23_ (100–1000 ms), corresponding to bound water, immobile water, and free water, respectively [38,39]. When comparing the treated samples with fresh ones (CK-0 group), there was no significant change in the T_21_ relaxation peaks of satsuma mandarins in each treatment group. The T_23_ relaxation peak remained close to that of the CK-0 group. However, the CK-40 group, which represents the control after 40 d of storage, displayed a significantly reduced free water content in comparison to the CK-0 group. In general, throughout the storage duration, there was a slight increase in the content of bound and immobile water across all groups of satsuma mandarins, while the content of free water decreased. When compared with the CK group, the LF-AEMF treatment group decreased in the reduction of free water content in the fruit. This result is consistent with the previously noted changes in weight of the satsuma mandarins and suggests that the application of LF-AEMF technology can effectively mitigate water loss in the fruit, thereby preserving their freshness.

## 4. Conclusions

In this study, we assessed the impact of LF-AEMF treatment at different strengths on various quality attributes of the satsuma mandarin, including appearance, flavor, and moisture content. The research results indicate that under the fridge conditions, the application of LF-AEMF treatment can effectively slow down the weight loss and respiration of satsuma mandarin, reduce sugar consumption, and thereby inhibit the deterioration of the quality of this fruit. During storage, the H-LF-AEMF treatment demonstrated superior efficacy in retarding the increase in MDA content compared with the other three groups, indicating that this treatment can effectively alleviate the oxidation of the fruit. Sensory attributes such as visual appearance, odor, sweetness, and juiciness of satsuma mandarins were more acceptable in LF-AEMF-treated groups after 40 days of storage. Additionally, the results of PAL activity indicate that the H-LF-AEMF treatment can maintain a higher level of PAL activity, which may contribute to enhancing the disease resistance of satsuma mandarins. The results of flavor and water migration indicate that, compared with the CK group, the LF-AEMF treatment can better maintain the original flavor of satsuma mandarins and inhibit the reduction of their free water content.

Overall, this study not only emphasizes the necessity of quantifying LF-AEMF intensity to achieve ideal preservation efficiency but also provides a promising strategy for extending the shelf life of citrus fruits. The applicability of this technology may also be extended to other fruits and vegetables suitable for long-term storage. Meanwhile, it is necessary to regulate more electromagnetic field parameters to further verify its preservative effect, which will be gradually demonstrated in subsequent studies.

## Figures and Tables

**Table 1:** Performance of electronic nose sensors.

Sensor Name	Performance Characteristics
W1C	Sensitive to aromatic compounds
W5S	High sensitivity, mainly detecting nitrogen oxides
W3C	Sensitive to ammonia and aromatic compounds
W6S	Sensitive to hydrogen
W5C	Sensitive to alkanes, aromatic compounds, and weakly polar compounds
W1S	Mainly detects alkanes, aromatic compounds, and weakly polar compounds
W1W	Sensitive to inorganic sulfides and terpenes
W2S	Wide range of substances, mainly detects ethanol and some aromatic compounds
W2W	Sensitive to aromatic compounds and sulfur-containing organic compounds
W3S	Sensitive to alkanes

## Data Availability

The original contributions presented in this study are included in the article. Further inquiries can be directed to the corresponding author.
